# The Tip-of-the-Tongue Phenomenon: Cognitive, Neural, and Neurochemical Perspectives

**DOI:** 10.3390/biomedicines14020269

**Published:** 2026-01-25

**Authors:** Chenwei Xie, William Shiyuan Wang

**Affiliations:** 1 Research Centre for Language Cognition and Neuroscience, Department of Language Science and Technology, The Hong Kong Polytechnic University, Hunghom, Hong Kong 999077, China; 2 Department of Electronic Engineering, The Chinese University of Hong Kong, Sha Tin, Hong Kong 999077, China; wsyw@cuhk.edu.hk

**Keywords:** tip-of-the-tongue, lexical retrieval, GABA, glutamate, excitation–inhibition balance, neuroimaging, proton magnetic resonance spectroscopy, aging

## Abstract

The tip-of-the-tongue (TOT) phenomenon is a transient state in which speakers momentarily fail to retrieve a known word despite preserved semantic knowledge and a strong sense of imminent recall. This review integrates cognitive and neural evidence with emerging neurochemical perspectives to develop a comprehensive biomedical framework for word-finding failures. Cognitive models of semantic–phonological transmission and interloper interference have been refined through structural, functional, and metabolic imaging to elucidate the mechanisms underlying TOT states across the lifespan. Functional neuroimaging implicates a left-lateralized fronto-temporal network, particularly the inferior frontal gyrus (IFG), anterior cingulate cortex (ACC), and temporal pole, in retrieval monitoring and conflict resolution. Structural MRI and diffusion imaging link increased TOT frequency to reduced integrity of the arcuate and uncinate fasciculi and diminished network efficiency. Proton magnetic resonance spectroscopy (^1^H-MRS) introduces a neurochemical dimension, with studies of related language tasks implicating lower γ-aminobutyric acid (GABA) and altered glutamate concentrations in frontal and temporal cortices as potential contributors to slower naming and heightened retrieval interference. Together, these findings converge on a model in which transient lexical blocks arise from local disruptions in excitation–inhibition (E/I) balance that impair signal propagation within language circuits. By uniting behavioral, neuroimaging, and neurochemical perspectives, TOT research reveals how subtle perturbations in cortical homeostasis manifest as everyday cognitive lapses and highlights potential biomedical strategies to maintain communicative efficiency across the lifespan.

## 1. Introduction

The tip-of-the-tongue (TOT) phenomenon, also referred to as lethologica, is a transient metacognitive state in which an individual is temporarily unable to retrieve a known word, despite a strong feeling of imminent recovery [1,2,3]. This everyday experience, though typically benign in younger adults and more frequent in older adults [4], is a highly informative window into the mechanisms of lexical access and self-monitoring within the human language system. In its classic behavioral manifestation, the speaker experiences partial phonological access, such as knowing the initial letter or the number of syllables, while still being unable to articulate the full target word [5]. Because this state sits at the boundary between successful and failed retrieval, its analysis yields insight into the fine-grained processes that transform conceptual activation into phonological output.

Systematic research on TOTs began with the landmark experiments of Brown and McNeill [5], who demonstrated that the phenomenon could be reliably elicited under laboratory conditions and is distinct from simple forgetting [6]. Since then, lexical retrieval has been modeled as a multi-stage process comprising semantic activation, lemma selection, and phonological encoding [7,8]. Failures at the latter stage, in which transmission between semantic and phonological nodes is momentarily disrupted, are thought to give rise to the TOT state. Competing theoretical accounts, including the transmission-deficit model, blocking/interference model, and metacognitive monitoring model, emphasize different loci of failure but converge on the view that TOTs reflect incomplete activation cascades within lexical networks [6,9,10].

Neuroimaging research has identified a distributed fronto-temporal circuit underlying lexical retrieval and word selection. In particular, the left inferior frontal gyrus (IFG), anterior cingulate cortex (ACC), and temporal pole become more active during TOT events compared with effortless retrieval [11,12,13,14]. Although cognitive and neuroimaging studies have advanced understanding of the TOT phenomenon, its neurochemical underpinnings remain to be empirically examined. Advances in proton magnetic resonance spectroscopy (^1^H-MRS) now allow in vivo quantification of cortical metabolites such as γ-aminobutyric acid (GABA) and glutamate, the principal inhibitory and excitatory neurotransmitters [15]. These molecules jointly ensure the balance of excitatory–inhibitory activity [16,17], which is crucial for selective attention and language control. Perturbations of this balance, which become more frequent in later adulthood, may underlie the transient failures of lexical selection that define TOT states.

The integration of neurochemical imaging with cognitive models represents an important step toward a comprehensive account of language production as a multilevel phenomenon that encompasses cognitive representations, neural dynamics, and metabolic regulation. While previous reviews have summarized the phenomenology and cognitive accounts of the TOT phenomenon [1,18], the present review advances the literature by explicitly integrating (i) cognitive models of TOT, (ii) converging multimodal neuroimaging evidence on the implicated language and control networks, and (iii) a neurochemical framework centered on E/I balance and neuromodulatory influences. By linking these levels of description, we aim to provide an interdisciplinary, mechanistically grounded account and to outline concrete, testable directions for future studies combining TOT paradigms with neurochemical measurement approaches (e.g., ^1^H-MRS and functional MRS).

## 2. Cognitive Models Underlying TOT States

Empirical investigation of the TOT state relies on standardized behavioral paradigms that elicit and quantify transient word-finding failures under controlled conditions. The definition-naming task remains the most widely used laboratory paradigm. Adapted from the seminal work of Brown and McNeill [5], participants read definitions of low-frequency words (e.g., “a building for aircraft storage”) and indicate whether they know, do not know, or are in a TOT state. Confirmed TOTs are followed by prompts for the target word, allowing measurement of TOT frequency, resolution latency, and accuracy of partial phonological information (e.g., initial letter, syllable count). Proper-name and face-naming paradigms extend this method by presenting photographs or descriptions of well-known people, landmarks, or objects [11,19]. These yield consistently higher TOT rates and are particularly sensitive to age-related and pathological retrieval deficits. Cueing and priming manipulations, which provide semantic, phonemic, or orthographic cues, probe the dynamics of lexical retrieval and the efficiency of inhibitory control mechanisms [9,20,21]. This methodological rigor underpins the translation of a subjective linguistic experience into a quantifiable biomarker of neural integrity and a means to test hypotheses derived from cognitive models of TOT.

### 2.1. Two-Stage and Interactive Activation Models

Foundational psycholinguistic frameworks depict lexical retrieval as proceeding through at least two phases: semantic/lemma selection, where conceptual and syntactic representations are accessed, and phonological encoding, where the word’s sound form is retrieved and prepared for articulation [22,23]. Evidence from naming and priming studies demonstrates that semantic activation can occur without corresponding phonological completion, supporting the two-stage conceptualization [24]. For example, speakers can accurately describe a target’s meaning or initial letter during a TOT, highlighting successful lemma activation but failed phonological retrieval [25].

Subsequent interactive activation models extend this account by incorporating bidirectional connections between semantic, lexical, and phonological nodes [26,27]. These models explain how activation can flow backward from partial phonological cues to facilitate recovery, consistent with the observation that prompting with the first sound or syllable often resolves a TOT episode [9,20]. The degree of connectivity between nodes and the efficiency of spreading activation jointly determine whether activation crosses the retrieval threshold required for articulation.

### 2.2. Transmission-Deficit Model

The Transmission-Deficit Model, proposed by Burke and colleagues, remains the most influential explanation of TOT frequency and distribution [9]. Similar to two-stage and interactive activation models, it posits that TOTs occur when the transmission of activation from semantic to phonological nodes is too weak to reach threshold. Over the lifespan, connection strengths may degrade due to reduced neural efficiency or plasticity, thereby increasing the likelihood of activation failure despite preserved semantic representations [28,29]. Empirical support for the Transmission-Deficit Model comes from aging studies showing that older adults experience more phonological errors but not greater semantic errors in picture naming tasks, implying a specific breakdown at the phonological interface [24,30]. This model also provides a viable framework for integrating neurochemical hypotheses, since weakened synaptic transmission could arise from altered neurotransmitter availability, particularly within systems regulating excitatory and inhibitory signaling.

### 2.3. Blocking/Interference Model

An alternative explanation emphasizes competitive interference during lexical selection rather than connection weakness. According to the blocking/interference model, TOTs result when a semantically or phonologically related interloper gains temporary advantage within the retrieval network, effectively blocking access to the intended word [31]. Experimental paradigms using semantic competitors or near neighbors (e.g., harp when trying to name lyre) increase TOT probabilities [32]. Neuroscientific evidence supports this mechanism: activation of the left IFG and ACC during TOTs reflects heightened selection demands and conflict monitoring [11,12]. Cognitive control is required to inhibit incorrect interlopers and sustain attention until the correct phonological form emerges [33,34]. In this context, GABAergic inhibition (addressed in Section 3.3) may serve as the neurochemical substrate enabling suppression of interlopers, while glutamatergic excitation drives the activation of target representations.

### 2.4. Metacognitive Monitoring Model

TOTs are not merely retrieval failures but also phenomenological states in which individuals are consciously aware that they know the desired word [18]. Models of metacognitive monitoring conceptualize the TOT state as a product of the individual’s internal assessment of memory strength signals [18]. The feeling of knowing arises when partial cues, such as semantic familiarity and contextual fit, exceed a subjective threshold, prompting the conscious recognition of imminent retrieval [11]. Neuroimaging research has implicated the anterior prefrontal cortex and ACC in such monitoring functions [35]. This view highlights the TOT as a bridge between cognitive control and consciousness, emphasizing higher-order assessment of retrieval states. Importantly, metacognitive awareness may influence retrieval persistence; individuals with stronger TOT-related familiarity signals are more likely to continue searching and eventually succeed [36]. Moreover, EEG research has indicated that alpha expression and suppression are observed broadly across centro-parietal scalp electrodes and disappear immediately upon presentation of the resolving feedback, further indicating a spontaneously occurring, conscious, and highly motivating goal-directed internal metacognitive state [37].

### 2.5. Integrative and Hybrid Models

**Figure 1:**
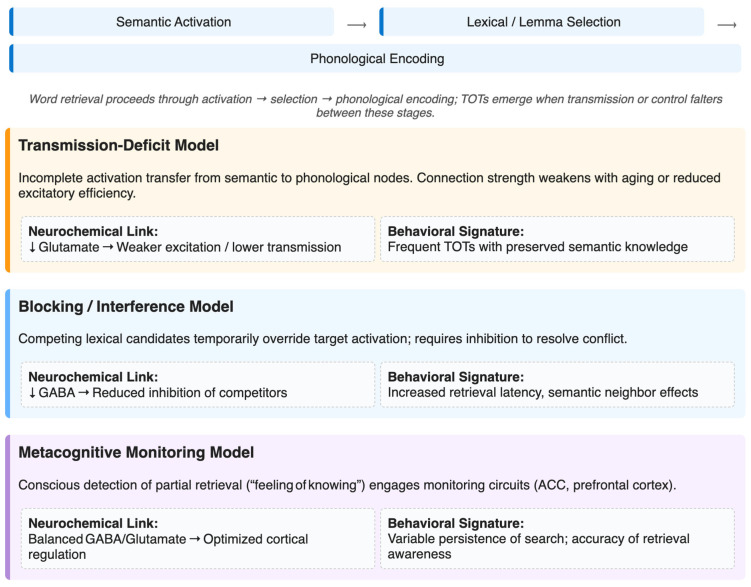
Cognitive models and proposed neurochemical mechanisms underlying the TOT phenomenon. Lexical retrieval proceeds through sequential activation of semantic, lexical–lemma, and phonological representations, as indicated schematically by the right-pointing arrows. TOT states emerge when transmission or control processes falter at specific stages. The Transmission-Deficit Model (orange panel) explains TOTs as weakened activation between semantic and phonological nodes. The Blocking/Interference Model (blue panel) attributes TOTs to competition among closely related lexical candidates resulting from insufficient inhibitory control. The Metacognitive Monitoring Model (violet panel) emphasizes awareness of partial retrieval signals and their regulation by anterior cingulate and prefrontal networks. The neurochemical associations illustrated here (e.g., glutamatergic excitation, GABAergic inhibition, and balanced E/I states) are proposed integrative hypotheses linking cognitive models to underlying cortical physiology. Downward arrows indicate reduced glutamate and GABA concentrations, while right-pointing arrows depict the potential cognitive and behavioral impacts. Together, these perspectives highlight how cognitive, neural, and neurochemical dynamics may jointly determine whether lexical access resolves successfully or stalls in a transient TOT state. This figure is drawn by Canva (https://www.canva.com/design/DAG_DNWEOcU/mpccnglDYbefEUotJbaenQ/edit?utm_content=DAG_DNWEOcU&utm_campaign=designshare&utm_medium=link2&utm_source=sharebutton, accessed on 23 October 2025).

Recent theoretical developments advocate hybrid frameworks that integrate elements of transmission-deficit, competition, and metacognitive monitoring accounts [6]. These models conceptualize TOTs as the outcome of both bottom-up activation failure (weakened semantic–phonological transmission) and top-down regulatory processes (ineffective inhibition or premature selection termination). In essence, the TOT is viewed as a systems-level metastable state that reflects dynamic interactions among multiple control mechanisms. This integrative perspective naturally accommodates neurochemical modulation of retrieval, as changes in GABAergic or glutamatergic tone may influence both activation strength and inhibitory efficacy. Weak excitation would parallel the reduced synaptic transmission proposed in the Transmission-Deficit Model, whereas impaired inhibition could account for blocking or interference dynamics. Figure 1 presents an integrated schematic of the major cognitive models of TOT and their proposed biochemical correlates. This convergence of cognitive models, neurochemical mechanisms, and empirical evidence forms the conceptual foundation for the next section’s discussion of neuroimaging and spectroscopy-based insights.

## 3. Neural and Neurochemical Perspectives on the TOT Phenomenon

The TOT phenomenon can be conceptualized as a systems-level event arising from interactions among distributed neural networks and modulating neurochemical processes. Although direct neurochemical investigations of TOT are lacking, substantial neuroimaging evidence delineates its cortical dynamics [19,38,39,40,41]. Findings from spectroscopy and related language tasks, including verbal fluency and semantic processing tasks, provide theoretical support for how E/I balance may shape word-finding success [33,42,43,44,45]. This section reviews neural evidence and integrates emerging neurochemical perspectives to outline a comprehensive framework for understanding the biological mechanisms underlying TOT.

### 3.1. Functional Neuroimaging Evidence

During TOT episodes, greater hemodynamic activity is consistently observed in the left IFG, ACC, and left middle and superior temporal gyri [11,12,21,38,39,46]. The IFG is implicated in the controlled selection and inhibition of competing semantic or lexical representations [47], whereas the temporal lobe underpins the storage and retrieval of lexical–semantic knowledge [48]. Enhanced activation of the ACC during TOT has been interpreted as reflecting conflict monitoring and error detection, consistent with its role in signaling a mismatch between intended and retrieved information [49]. Together, these regions constitute a fronto-temporal feedback loop that regulates the transition from semantic retrieval to articulatory planning.

An essential methodological challenge has been to disentangle retrieval effort from TOT-specific activation patterns. Comparative fMRI paradigms contrasting TOT states with both successful and failed retrievals show that activation in ACC and right prefrontal regions increases with retrieval difficulty and exhibits a distinct activation pattern during TOT [12]. In contrast, purely successful retrievals without TOT awareness elicit reduced or absent frontal activation, underscoring that conscious metacognitive monitoring distinguishes the TOT condition from simple retrieval failure [46]. Electrophysiological and magnetoencephalographic data reveal that the engagement of the left inferior frontal and anterior cingulate regions is temporally delayed in TOT states relative to fluent retrieval, with activation shifting from early (≈300–500 ms) to later (≈600–800 ms) post-stimulus intervals [14,50]. This temporal delay likely represents prolonged conflict resolution and reinstatement attempts, which mirror the search phase experienced subjectively. Such findings support models positing that TOTs emerge from recurrent activation cycles within the language network rather than a single, failed transmission.

Notably, the amplitude and timing of neural signals correspond well with known effects of inhibitory (GABAergic) and excitatory (glutamatergic) modulation at the cortical network level [15]. For instance, local GABA concentration within the ACC correlates negatively with blood oxygen level-dependent (BOLD) activation magnitude and positively with efficiency in detecting and monitoring response conflict [51]. Conversely, glutamatergic signaling supports excitatory drive within primary visual cortex, fostering coherent visual activation [52]. Although not directly testing lexical retrieval, these relationships, quantifiable by ^1^H-MRS, may provide a mechanistic bridge between functional neuroimaging and neurochemical data underpinning the TOT mechanism, which will be elaborated in Section 3.3.

### 3.2. Structural Correlates of Lexical Retrieval

While functional imaging delineates the dynamic engagement of cortical regions during TOT events, structural neuroimaging and connectivity analyses reveal the anatomical substrates that enable, and sometimes constrain, lexical retrieval. Morphometric studies show that performance on word-retrieval and naming tasks correlates with gray-matter density and cortical thickness in language-related regions, particularly the left IFG, middle temporal gyrus (MTG), and temporal pole [13,53,54]. Individuals who report frequent TOTs tend to exhibit reduced gray-matter volume or cortical thinning in these same regions [55]. Such findings indicate that structural integrity of the semantic–phonological interface supports efficient transmission of activation across lexical levels, consistent with the Transmission-Deficit Model.

On the other hand, language retrieval depends on rapid, bidirectional information transfer between temporal representations of meaning and frontal articulatory planning. DTI research has elucidated the critical white-matter fiber tracts mediating these exchanges, most prominently the arcuate fasciculus (AF), uncinate fasciculus (UF), and inferior fronto-occipital fasciculus (IFOF) [56,57,58,59,60]. The dorsal AF connects posterior temporal regions with inferior frontal cortex, supporting phonological and articulatory encoding [61,62,63], whereas the ventral UF links anterior temporal semantic regions with orbitofrontal and ventral prefrontal regions involved in retrieval monitoring and evaluative control [64].

Empirical studies show that lower fractional anisotropy (FA), which reflects compromised fiber organization or myelination within fronto-temporal language tracts, correlates with worse performance on verbal fluency and confrontation naming tasks [65,66]. In older adults, microstructural deterioration of the left UF (which is one of the last white-matter tracts to mature in the human brain) in particular predicts failures of proper name retrieval [67]. These findings support the interpretation that TOTs reflect inefficient conduction or diminished signaling along fronto-temporal pathways, paralleling reduced effective excitation proposed in neurochemical accounts. Furthermore, diffusion-based measures of microstructural integrity have been shown to correlate with glutamate concentrations in adjacent cortical regions [68,69], supporting the view that structural and neurochemical organization co-evolve within the cortical networks underlying language processing.

### 3.3. Neurochemical Perspectives: GABA and Glutamate Contributions

Although converging neurochemical findings have not directly examined TOT states, they nonetheless imply that TOTs may arise from subtle perturbations in the cortical E/I balance. Word retrieval depends on coordinated interactions within a distributed fronto-temporal network, including the left IFG, ACC, insula, and temporal cortex [8,70,71]. Within these regions, cortical signaling stability is governed by the dynamic interplay between GABA, the brain’s primary inhibitory neurotransmitter, and glutamate, its principal excitatory counterpart. Alterations in this E/I balance have been linked to inefficiencies in neural selection and suppression processes [72], providing a biochemical framework for understanding TOT experiences.

#### 3.3.1. GABAergic Modulation of Lexical Retrieval Inhibition

Inhibitory control, likely mediated by GABAergic mechanisms [73], plays a crucial role in lexical selection by suppressing activation of irrelevant or competing words during retrieval [74]. In language production tasks, excessive competition among phonologically or semantically related interlopers can delay access to a target word, which is a cognitive hallmark of the TOT experience [32]. Neuroimaging and neurophysiological studies have shown that the left IFG and ACC mediate this conflict resolution process [21,75]. Both regions are rich in GABAergic interneurons that precisely regulate pyramidal cell excitability and network balance [76], and both regions exhibit an age-related decline [77]. A reduction in local GABA concentration may impair this inhibitory filtering, leading to persistent activation of non-target competitors and difficulty completing word retrieval even when semantic access is intact [78,79].

^1^H-MRS studies provide direct, non-invasive quantification of regional GABA levels at rest or during task-related modulation [73,80]. Findings from such studies demonstrate that higher GABA concentrations within the left IFG or motor-speech areas correlate with faster naming, reduced interference, and greater articulatory efficiency [81,82]. In contrast, lower GABA levels have been associated with slower response times and increased susceptibility to visual competition effects [83], patterns reminiscent of TOT phenomena. Pharmacological observations further support this association: agents that potentiate GABA activity, such as benzodiazepines, are known to impair memory encoding, verbal fluency, and word-retrieval accuracy, producing transient effects analogous to incomplete lexical activation [84,85,86]. Although MRS and TOT paradigms have not yet been directly combined, this converging evidence suggests that individual variability in cortical GABA concentration may predispose some speakers to more frequent or prolonged TOT episodes.

#### 3.3.2. Glutamatergic Contribution to Lexical Activation and Propagation

If GABA constrains competing representations, glutamate or Glx (glutamate + glutamine) likely provides the excitatory drive [87], which activates semantic and phonological nodes within the language network [74]. Efficient propagation of activation through cortico-temporal connections, particularly within the middle and superior temporal gyri [88], relies on glutamatergic neurotransmission in these auditory–language cortices [89]. Dysregulated or inefficient glutamatergic signaling may weaken transmission between semantic and phonological processing stages, leading to the characteristic feeling of knowing but being unable to articulate the word [19,90].

Individual-difference analyses in cortical glutamate add further nuance, especially within the left temporal lobe and hippocampus, and predict verbal memory and naming performance [91,92]. Elevated glutamate correlates with superior learning and faster recall, whereas lower levels correspond to delayed retrieval [93]. Combined MRS-fMRI approaches reveal that regions with higher glutamatergic availability show stronger hemodynamic responsiveness during verbal generation tasks [94,95]. This supports a direct coupling between excitatory neurochemistry and BOLD activity underlying lexical access. These results emphasize the translational potential of MRS-based neurometabolic indices to identify micro-variability in word retrieval performance among healthy individuals and to characterize language inefficiency in clinical aging populations [96].

#### 3.3.3. Excitation–Inhibition Balance Insights

**Figure 2:**
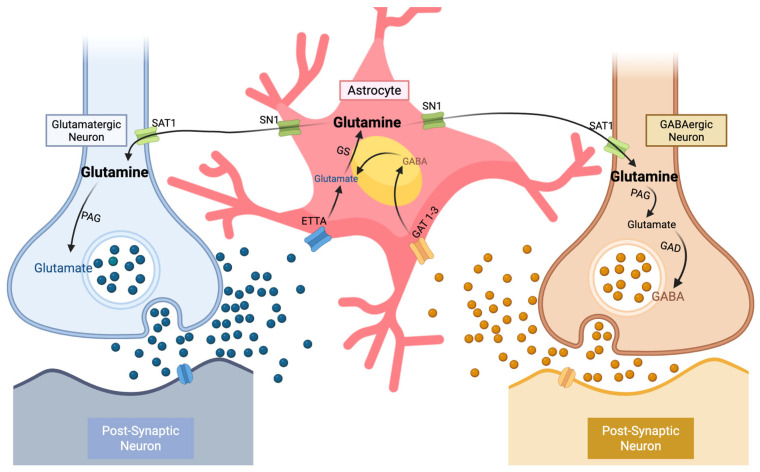
Schematic representation of the glutamine–glutamate/GABA cycle, adapted from Newsholme, Diniz [100]. (1) Within glutamatergic and GABAergic neurons, the excitatory neurotransmitter glutamate and the inhibitory neurotransmitter GABA are released into their respective synaptic clefts to signal postsynaptic targets. (2) After neurotransmission, synaptic glutamate and GABA are rapidly removed from the extracellular space by excitatory amino acid transporters (EAATs) and GABA transporters (GAT-1 and GAT-3) located on surrounding astrocytic membranes. (3) Within astrocytes, glutamate is amidated by glutamine synthetase (GS) to form glutamine, while GABA is metabolized via the tricarboxylic acid (TCA) cycle and subsequently converted to glutamine through GS activity. (4) Glutamine produced in astrocytes exits to the extracellular space through system N transporter 1 (SN1) proteins and diffuses back to presynaptic neurons, where it is taken up by diamine acetyltransferase (SAT1). (5) Inside presynaptic terminals, glutamine is reconverted to glutamate by phosphate-activated glutaminase (PAG) and, in GABAergic neurons, further decarboxylated by glutamic acid decarboxylase (GAD) to regenerate GABA. Together, these astrocyte–neuron interactions sustain the continuous cycling of excitatory and inhibitory neurotransmitters, preserving cortical E/I homeostasis. Created in BioRender. XIE, C. (2026) https://app.biorender.com/profile/template/details/t-6970be9e5b80e52fa21598ae-glutamine-glutamategaba-cycle.

The co-modulation of GABA and glutamate is critical: excessive excitation without adequate inhibition may also hinder retrieval by amplifying neural noise and item competition [42,43]. This imbalance is particularly relevant to TOT states, where elevated glutamatergic activity relative to GABA can intensify interference from phonological or semantic interlopers, prolonging unresolved retrieval blocks and increasing TOT frequency. For example, Jung and colleagues [43] found that participants with higher relative glutamate concentrations (indicating lower E/I balance) in the left anterior temporal lobe exhibited greater behavioral disruption and altered task-evoked connectivity when semantic processing was perturbed by continuous theta-burst stimulation, highlighting increased vulnerability to competing representations. Moreover, human associative memories are stored in balanced E/I ensembles that lie dormant unless latent inhibitory connections are unmasked [17]. Therefore, optimal linguistic performance and the avoidance of TOT states likely arise from an optimal GABA–glutamate ratio enabling precise activation with controlled suppression. Figure 2 illustrates the canonical glutamine–glutamate/GABA cycle that links neurons and astrocytes in maintaining E/I balance. This biochemical loop is central to neurometabolic efficiency in language-related regions: astrocytes reclaim glutamate and GABA from the synaptic cleft, convert them into glutamine, and shuttle this precursor back to neurons for resynthesis of neurotransmitters. Disruptions at any step in this cycle may alter local neurotransmitter availability and, consequently, the temporal precision required for successful lexical retrieval. Beyond glutamate and GABA, cholinergic neuromodulation interacts with dopaminergic signaling and can regulate both glutamatergic excitation and GABAergic inhibition via neuronal and astrocytic mechanisms at the tripartite synapse, thereby fine-tuning cortical gain and potentially shaping vulnerability to lexical interference [97,98,99].

In summary, the TOT phenomenon can be conceptualized as a functional echo of transient neurochemical imbalances within the fronto-temporal language circuit. GABA provides the inhibitory precision needed to suppress lexical interlopers, whereas glutamate drives the excitatory activation necessary for successful access. A key limitation of the current literature is that, to our knowledge, no published ^1^H-MRS or functional MRS studies have directly measured GABA, glutamate/Glx, or related metabolites during experimentally elicited TOT states. Therefore, the proposed neurochemical interpretation of TOT should be regarded as a testable framework grounded in convergent findings from related domains (e.g., naming, semantic interference, and cognitive control tasks) rather than direct neurochemical evidence during TOT episodes. Future targeted spectroscopy studies in populations prone to TOT could decisively link metabolite concentrations with language performance, clarifying how the molecular architecture of cortical balance shapes everyday cognitive flexibility and word-finding behavior [101].

### 3.4. Multimodal Imaging Integrating Structure, Function, and Neurochemistry

The TOT phenomenon reflects transient disconnection between semantic–lexical and phonological–articulatory levels of word retrieval. Because these processes depend on both anatomical connectivity and neurochemical modulation, no single imaging modality can fully capture the underlying mechanisms. Recent multimodal approaches that combine diffusion-weighted MRI (DW-MRI), functional MRI (fMRI), and ^1^H-MRS enable the simultaneous assessment of structural pathways, functional dynamics, and neurotransmitter balance within the same individuals [102,103,104,105,106].

#### 3.4.1. Microstructure–Function Correlations

Multimodal imaging shows that fronto-temporal white-matter integrity, particularly within IFOF, predicts the efficiency of BOLD activation and effective connectivity between the left IFG and MTG during lexical–semantic processing [107]. For instance, by combining the direct functional interactions between the nodes of the language networks with the fiber tracking data, it demonstrates that the functional interactions between brain regions during language processing are mediated by specific white-matter pathways in dorsal stream (involving phonological processing) and ventral stream (involving semantic processing) [108]. Individuals with greater FA and more coherent tract microstructure are associated with increased BOLD responses during working memory, visual processing and motor tasks [109,110,111], and this positive association is reversed in older adults [112,113].

Furthermore, the structural integrity of cortical pathways sets the physical limits on how efficiently excitatory and inhibitory activity can propagate through linguistic circuits [69,114]. Degradation of white-matter fibers may limit effective glutamatergic transmission, whereas cortical thinning in inhibitory control regions could impair GABAergic modulation of competing representations. Conversely, compromised neurochemical efficiency, such as reduced concentrations of excitatory or inhibitory metabolites, can disrupt homeostatic E/I regulation, leading to maladaptive changes in myelination and large-scale connectome organization [115,116]. Although these studies do not examine the functional and anatomical connectivity of TOT, such relationships may suggest that TOT susceptibility is related to inefficiencies in both physical conduction pathways and dynamic information exchange within the language network.

#### 3.4.2. Neurochemical–Functional Integration

^1^H-MRS studies reveal that GABA and glutamate concentrations within the IFG and temporal cortices shape functional performance [73,117]. Higher inhibitory tone (GABA) in IFG is associated with smaller cerebral blood flow [118], consistent with more efficient signal-to-noise control, which may contribute to improved lexical selection accuracy. Conversely, elevated glutamatergic excitation in the anterior and middle temporal areas [48] may predict stronger semantic activation and faster naming responses, consistent with findings linking regional glutamate concentrations to individual differences in linguistic performance [119]. By acquiring MRS and functional data within a single imaging session, it is possible to correlate metabolite concentrations with neural activation and connectivity [120,121]. For instance, regional glutamate and GABA levels in the posteromedial cortex positively and negatively predict intrinsic functional connectivity strength of the default mode network during rest [122]. These multimodal associations underscore that BOLD activity is shaped not only by pathway integrity but also by the local neurochemical environment governing E/I balance [15].

Traditional MRS studies have been largely static, measuring metabolite concentrations only at rest. In contrast, emerging functional or dynamic MRS (fMRS) techniques permit time-resolved assessment of neurotransmitter fluctuations during cognitive tasks [45,123]. Applying fMRS to TOT paradigms could reveal how real-time changes in GABA and glutamate accompany retrieval attempts, self-monitoring, and successful resolution. When combined with fMRI, this temporal precision would help delineate the neurochemical dynamics of language control, advancing causal models of how transient shifts in the cortical E/I balance generate the subjective experience of imminent yet inaccessible recall that defines the TOT phenomenon.

Emerging techniques extend these integrations further. High-resolution MR spectroscopic imaging (MRSI) now enables full-brain, millimeter-scale metabolite mapping, including the left perisylvian cortex, through accelerated spatial–spectral acquisition methods [124,125,126]. In parallel, connectome-based predictive modeling relates large-scale functional and structural connectivity patterns to behavioral phenotypes such as naming speed or TOT frequency [127,128]. Pairing task-related fMRI with real-time E/I ratio mapping may soon clarify how dynamic modulation of GABA and glutamate orchestrates shifts between successful retrieval and TOT states. In addition, multimodal fusion algorithms, such as joint independent component analysis and Bayesian connectivity modeling, can integrate structural, functional, and potentially metabolic datasets within a unified analytical framework [102,129,130,131,132]. These methods promise a genuinely holistic characterization of lexical retrieval as a process grounded in both anatomical and neurochemical balance.

## 4. Aging and Cognitive Decline in TOT Phenomena

Exploration of TOT states exemplifies a model for understanding everyday cognitive lapses as a biochemically constrained systems phenomenon. It demonstrates how subtle alterations in cortical neurotransmitter balance can transiently disrupt behavior and how restoring neurochemical equilibrium may support cognitive performance and overall well-being in older or neurologically vulnerable populations [133].

### 4.1. Lifespan Trends in TOT Frequency

Across adulthood, the TOT experience increases reliably in frequency, even when general intelligence or vocabulary size remains stable [3,4]. Behavioral studies show that older adults experience roughly twice as many TOTs as young adults, particularly for low-frequency words and proper names [9,134]. Despite this increase, semantic accuracy and comprehension are typically preserved, indicating that the deficit lies at the phonological encoding stage rather than in semantic memory per se [30]. Moreover, longitudinal and lifespan studies indicate that age-related increases in TOT states reflect selective inefficiency in lexical–phonological retrieval mechanisms, rather than a manifestation of generalized cognitive decline [135,136,137]. The escalation of TOTs often precedes measurable losses in other cognitive domains, positioning it as an early behavioral indicator of changes in the language control network [135,138].

From a cognitive modeling standpoint, as discussed in Section 2, the increased incidence of TOT states with advancing age can be accounted for by complementary mechanisms. Within the transmission-deficit framework, aging weakens the connections between semantic and phonological representations [28], potentially reflecting synaptic deterioration or reduced neurotransmitter efficacy. Blocking/interference models, in turn, emphasize an age-related decline in the ability to suppress competing lexical interlopers, thereby increasing interference and prolonging retrieval attempts [31]. Monitoring-adjustment accounts propose that older adults alter the metacognitive thresholds used to classify a state as “I know it,” leading to more frequent subjective reports of TOT experiences [18]. Moreover, aging is often accompanied by prolonged search durations following a TOT episode, suggesting persistence and the engagement of additional cognitive resources [139]. This compensatory behavior aligns with fMRI evidence showing increased frontal activation during verbal retrieval in older adults, widely interpreted as the adaptive recruitment of executive control circuits [140,141].

### 4.2. Structural and Functional Correlates of Age-Related TOTs

Neuroimaging studies reveal that age-related increases in TOTs correspond to structural and functional alterations in language-related networks. Voxel-based morphometry and DTI analyses demonstrate age-related cortical thinning in the left inferior and superior frontal gyri and reduced white-matter integrity (lower FA) along the fronto-temporal AF [142,143]. Importantly, semantic knowledge appears preserved in aging despite reduced activation in frontotemporal control regions, indicating that phonological or executive encoding mechanisms are selectively affected [24,144]. At the cellular level, decreases in cortical thickness likely reflect reduced dendritic arborization and synaptic density, leading to weakened excitatory connectivity, a process paralleled by findings of lower cortical glutamate concentrations with aging [69,145]. These microstructural degradations may predict higher TOT frequency and slower word retrieval.

Functionally, older adults show heightened activation in bilateral ACC and frontal regions during naming and TOT trials [146]. This pattern differs from the more focal left-dominant activation seen in younger individuals, suggesting a shift toward bilateral recruitment and reduced neural specificity and co-existing compensation and reorganization mechanisms [147,148]. Compensatory engagement of homologous cortical networks may transiently sustain cognitive performance but at the cost of heightened metabolic demand and neural noise, paralleling age-related reductions in GABAergic inhibitory control [149,150,151]. Together, alterations in axonal integrity and frontal neurometabolic profiles suggest a shared degenerative trajectory underlying lexical retrieval difficulties.

### 4.3. Neurochemical Aging: Altered Excitation–Inhibition Balance

Aging is accompanied by measurable changes in the cortical E/I balance, particularly involving GABA and glutamate, both quantifiable in vivo with proton MRS. Studies consistently report declining GABA concentrations in frontal and temporal cortices across adulthood [77,90,152]. Given that GABA mediates inhibitory control, such reductions are associated with behavioral disinhibition and impaired response inhibition [153], a pattern that may also underlie increased retrieval interference and, consequently, more frequent TOT experiences in older adults. Moreover, declining GABA correlates with poorer performance on tasks requiring cognitive inhibition and attentional control [154,155], suggesting that age-related reductions in inhibitory neurotransmission may broadly compromise the regulation of lexical activation and selection during word retrieval.

Glutamate levels also change with age, although findings are more variable. While some studies report mild cortical glutamate reductions linked to slower semantic and verbal fluency performance [44,87], others show relative preservation of glutamatergic tone in frontal white matter and compensatory increases in the anterior cingulate and right auditory cortices [156,157,158], possibly reflecting region-specific upregulation of the glutamate–glutamine cycle that helps sustain excitatory signaling in early aging. The combined decline in GABAergic and possibly glutamatergic function with age reduces the precision of neural signaling, increases background noise, and weakens semantic–phonological transmission [159], representing a neurochemical analog of the Transmission-Deficit account proposed in cognitive models.

### 4.4. Multimodal Evidence of Aging Effects

Emerging multimodal imaging approaches strengthen the link between neurochemical and structural changes underlying age-related vulnerability to TOT states. In a cohort of healthy male volunteers, Arrubla and colleagues [68] reported a negative correlation between glutamate concentration measured by MRS in the posterior cingulate cortex and local mean diffusivity, indicating that higher glutamatergic levels are associated with greater microstructural integrity. It is therefore plausible that neurotransmitter decline and microstructural deterioration co-occur within the same network nodes. Such overlap would support a systems-level interpretation in which disruption of the E/I balance and diminished axonal conduction efficiency jointly destabilize lexical processing loops.

Functional MRS studies further demonstrate activity-dependent modulation of GABA and glutamate during various visual, motor, memory, inhibition, and language tasks [33,160,161,162,163,164]. Young adults increase glutamate turnover and suppress GABA to boost activation during retrieval [118,165], whereas older adults show blunted neurochemical modulation [166]. The diminished flexibility of the E/I system may therefore underlie both reduced retrieval efficacy and decreased capacity to recover from a TOT episode.

## 5. Clinical and Biomedical Implications

The convergence of cognitive, structural, and neurochemical evidence on the TOT phenomenon extends its relevance beyond theoretical psycholinguistics into clinical and translational neuroscience. Persistent or exaggerated TOT experiences may indicate subtle inefficiencies in the neural systems subserving word retrieval, inefficiencies that can precede measurable cognitive decline [9,19,135]. Consequently, characterizing the biomedical signatures of TOT has the potential to inform both diagnostic biomarkers and therapeutic interventions aimed at preserving language integrity across aging and disease.

### 5.1. TOT as a Cognitive Biomarker of Neural Efficiency

The frequency of TOT states and the latency with which they are resolved serve as sensitive behavioral indices of lexical retrieval efficiency, indexing the functional integrity of frontotemporal control and phonological access networks [38,39]. Integrating these behavioral indices with neurochemical and structural imaging could enhance their diagnostic specificity [69,167]. For instance, elevated TOT rates accompanied by reduced GABA or glutamate concentrations and decreased AF integrity may constitute a promising marker of early network degradation before the onset of overt dementia. Such multimodal profiling aligns with the emerging framework of “neurochemical connectomics,” which conceptualizes brain connectivity in molecular terms by examining inter-regional covariation in neurochemical signals [168]. Although this approach has been largely driven by PET-based analyses, proton MRS can extend the same principle by relating regional concentrations of metabolites such as GABA and glutamate to variations in structural and functional connectivity.

In longitudinal contexts, tracking TOT frequency alongside MRS-derived E/I ratios can serve as an accessible, non-invasive measure of cortical health. Given that TOT tasks require minimal equipment and are adaptable across languages with appropriate norming, they are exceptionally suited for large-scale or clinical screening paradigms [135]. Biomarker frameworks following the “AT(N)X” model of Alzheimer pathology, where A = amyloid, T = tau, N = neurodegeneration [169,170,171,172], may soon include an “X” domain representing E/I equilibrium. Within this paradigm, elevated TOT rate combined with altered GABA–glutamate balance could signal early dysregulation in cortical processing prior to structural atrophy. Such an addition would bridge behavioral symptomatology with quantifiable biochemical and imaging metrics.

### 5.2. Neurochemical Targets for Pharmacological Modulation

Insights from GABAergic and glutamatergic contributions to TOT open promising avenues for neurochemical modulation of lexical retrieval. Pharmacological agents that normalize E/I balance may enhance language processing efficiency by stabilizing activation flow and suppressing excessive interference. While broad benzodiazepine-type modulation of GABAA receptors is associated with diffuse cognitive slowing and sedation [86], low-dose subtype-selective agents acting at the α2/α3 subunits can sharpen inhibitory precision without engaging sedative α1-pathways [173], thereby potentially enhancing lateral inhibition and improving lexical selection control. On the other hand, pharmacological agents that enhance glutamatergic transmission through NMDA-receptor co-agonist mechanisms, such as D-serine or the GlyT1 inhibitor sarcosine, have demonstrated significant improvements in learning, memory, and broader cognitive performance in both preclinical models and clinical populations [174]. Beyond direct neurotransmitter modulation, cholinergic and dopaminergic agents, long recognized for their influence on language and cognitive plasticity, may exert their therapeutic effects in part through indirect normalization of GABAergic and glutamatergic signaling [175,176]. However, these strategies must balance the risk of excitotoxicity and systemic side effects. Controlled pilot studies in healthy elderly participants could evaluate whether transient enhancement of fronto-temporal E/I balance reduces induced TOT frequency or latency.

### 5.3. Non-Pharmacological and Neurostimulation Approaches

Non-invasive brain-stimulation techniques, such as transcranial direct-current stimulation (tDCS) and theta-burst transcranial magnetic stimulation (TMS), modulate cortical excitability by altering E/I balance. Anodal tDCS and inhibitory theta-burst TMS applied to left frontal or temporal regions have been shown to transiently shift local GABA and glutamate levels, indicating dynamic changes in inhibitory-excitatory tone [33,177,178]. MRS showed that anodal tDCS over the left IFG significantly reduces GABA concentrations for weeks and was accompanied by greater naming improvements than sham stimulation [96]. In older adults and individuals with aphasia, repeated sessions of non-invasive brain stimulation, particularly anodal tDCS paired with speech-language therapy, can enhance cortical plasticity and strengthen synaptic efficiency within language networks [179,180]. Similarly, aerobic and cognitive training interventions promote synaptic plasticity and inhibitory balance, with exercise elevating GABA levels and improving executive control [181,182]. Together, these non-pharmacological strategies may therefore help mitigate TOT occurrences by restoring neurochemical balance and reinforcing the inhibitory control mechanisms that support efficient lexical retrieval.

Ultimately, integrating TOT metrics with neurochemical and structural measures could help pave the way for personalized cognitive chemistry profiles that link word-retrieval performance to underlying circuit physiology. At the same time, it should be emphasized that the proposition of TOT frequency or severity as a validated early clinical biomarker of neurodegenerative disease is not yet robustly supported by empirical evidence. Any biomarker-oriented interpretation will require longitudinal studies that track individuals over time and relate TOT measures to clinical outcomes and established disease markers (e.g., neuropsychological profiles, imaging and fluid biomarkers) and, when possible, clinicopathological confirmation. Contingent on such validation, each individual’s linguistic performance could be interpreted within their specific neurochemical context (e.g., E/I-related markers), helping to test whether retrieval inefficiency is more consistent with excitation instability, inhibition deficits, or connectional breakdown [101]. Tailored interventions, whether pharmacological, behavioral, neuromodulatory, or combined, could then be selected to target the dominant putative pathway of dysfunction.

## 6. Future Directions

### 6.1. Toward Integrated Cognitive–Neurochemical Models

Future research should aim to synthesize cognitive theories of lexical retrieval with quantifiable neurochemical dynamics. Integrating computational models of E/I dynamics and metabolic measurements [183] with in vivo connectivity analyses of semantic and phonological processing [184] would enable direct tests of how neurochemical balance shapes the frequency and latency of TOT events. New biophysical modeling approaches, such as dynamic causal modeling (DCM) integrated with MRS data, could capture how fluctuations in GABA and glutamate alter effective connectivity within fronto-temporal circuits during word retrieval [185]. Such efforts will transform the TOT phenomenon from a descriptive behavioral occurrence into a mechanistically grounded model of neurolinguistic control.

### 6.2. Artificial Intelligence and Computational Neurophenotyping

Machine-learning approaches further hold promise for identifying complex multivariate patterns across behavioral, structural, and neurochemical domains. Algorithms trained on high-dimensional multimodal data could classify individuals according to TOT risk profiles or predict retrieval efficiency from neurochemical and connectivity fingerprints [128]. Furthermore, connectionist simulations of lexical production incorporating weakened transmission strengths can reproduce TOT-like behaviors [27,186]; extending these to biologically informed excitatory and inhibitory networks could test E/I perturbation effects. Aligning these simulations with empirical MRS data would provide a computational testbed for exploring interventions, pharmacological or neurostimulatory, that restore optimal network dynamics.

### 6.3. High-Field and Next-Generation Spectroscopy

Advances in ultra-high-field magnetic resonance (≥7 T) have substantially increased the sensitivity and spectral resolution of MRS for detecting low-concentration metabolites and differentiating overlapping spectral peaks [187]. These capabilities allow simultaneous quantification not only of total GABA and glutamate but also of subcomponents such as glutamine, GABA+, and glutathione (GSH), offering a richer neurochemical profile of cortical metabolism [188]. Future TOT studies at high field could map spatially specific E/I ratios across multiple language regions, including the inferior frontal gyrus, superior temporal gyrus, and anterior temporal lobe, to determine whether individual differences in local metabolite distributions predict differential vulnerability to lexical retrieval failure. Combining spectroscopic imaging with functional activation paradigms will permit real-time assessment of neurochemical dynamics during attempted word recall.

## 7. Conclusions

The TOT phenomenon has evolved from a classic topic in cognitive psychology to a multidisciplinary subject bridging linguistics, neuroscience, and biomedicine. Once regarded merely as a fleeting lexical lapse, it can now be recognized as a window into the micro-dynamics of cortical excitation, inhibition, and communication efficiency that sustain human language. Cognitive frameworks, including transmission-deficit, blocking/interference, and metacognitive monitoring models, delineate the informational and control processes that collapse transiently during TOTs. Neuroimaging studies extend these theories to a distributed fronto-temporal network, coordinating semantic activation, lexical selection, and retrieval monitoring. Structural and connectivity analyses reveal that degradation of this network’s gray- and white-matter integrity directly predicts TOT frequency, particularly in aging populations.

At the biochemical level, ^1^H-MRS has introduced quantifiable markers of GABAergic inhibition and glutamatergic excitation, enabling a neurochemical interpretation of linguistic performance. Evidence increasingly supports that reduced inhibitory precision (low GABA) and weakened excitatory transmission (altered glutamate) may destabilize the semantic–phonological interface, resulting in the characteristic feeling of knowing but not retrieving. From a clinical viewpoint, the TOT offers a sensitive behavioral biomarker of cortical efficiency and a potential early indicator of neurocognitive vulnerability. Its quantifiable frequency can be paired with neurochemical and structural metrics to monitor the health of language networks across the lifespan. Looking forward, advances in high-field spectroscopy, dynamic multimodal imaging, and computational neuromodeling will enable fine-grained mapping of E/I balance during real-time language use. Integrating behavioral, structural, and neurochemical indicators will clarify how microscopic changes in brain neurochemistry produce macroscopic phenomena in cognition. Such progress heralds a new stage where the study of everyday cognitive experiences, like the TOT state, contributes directly to personalized neurocognitive medicine and a deeper understanding of the biological foundations of human communication.

## Data Availability

This review article does not create new data.

## Abbreviations

The following abbreviations are used in this manuscript:

^1^H-MRS	proton magnetic resonance spectroscopy
ACC	anterior cingulate cortex
AF	arcuate fasciculus
BOLD	blood oxygen level-dependent
DCM	dynamic causal modeling
DTI	diffusion tensor imaging
DW-MRI	diffusion-weighted magnetic resonance imaging
E/I	Excitation–inhibition
EAATs	excitatory amino acid transporters
EEG	electroencephalography
fMRI	functional magnetic resonance imaging
fMRS	functional magnetic resonance spectroscopy
GABA	γ-aminobutyric acid
GABA+	γ-aminobutyric acid plus co-edited macromolecule signal
GAD	glutamic acid decarboxylase
GAT	γ-aminobutyric acid transporter
Glx	glutamate + glutamine
GS	glutamine synthetase
GSH	glutathione
IFG	inferior frontal gyrus
IFOF	inferior fronto-occipital fasciculus
MRS	magnetic resonance spectroscopy
MRSI	magnetic resonance spectroscopic imaging
MTG	middle temporal gyrus
NMDA	N-methyl-D-aspartate
PAG	phosphate-activated glutaminase
PET	positron emission tomography
SAT1	spermidine/spermine N^1^-acetyltransferase 1
SN1	system N transporter 1
tDCS	transcranial direct-current stimulation
TCA	tricarboxylic acid (cycle)
TMS	transcranial magnetic stimulation
TOT	tip-of-the-tongue
UF	uncinate fasciculus
